# Assay‐agnostic spatial profiling detects tumor microenvironment signatures: new diagnostic insights for triple‐negative breast cancer

**DOI:** 10.1002/1878-0261.13515

**Published:** 2023-09-11

**Authors:** Colleen Ziegler, Alain Mir, Sangeetha Anandakrishnan, Patrick Martin, Elma Contreras, Isaiah Slemons, Barbara Witkowski, Chris DeSilva, Andrew Farmer, Semir Vranic, Zoran Gatalica, David Richardson, Dmitry N. Derkach

**Affiliations:** ^1^ bioSyntagma Tempe Arizona USA; ^2^ Takara Bio USA San Francisco California USA; ^3^ Caris Life Sciences Phoenix Arizona USA; ^4^ College of Medicine, QU Health Qatar University Doha Qatar

**Keywords:** immuno‐oncology, spatial profiling, triple‐negative breast cancer, tumor microenvironment

## Abstract

The role of the tumor microenvironment (TME) in immuno‐oncology has driven demand for technologies that deliver *in situ*, or spatial, molecular information. Compartmentalized heterogeneity that traditional methods miss is becoming key to predicting both acquired drug resistance to targeted therapies and patient response to immunotherapy. Here, we describe a novel method for assay‐agnostic spatial profiling and demonstrate its ability to detect immune microenvironment signatures in breast cancer patients that are unresolved by the immunohistochemical (IHC) assessment of programmed cell death ligand‐1 (PD‐L1) on immune cells, which represents the only FDA microenvironment‐based companion diagnostic test that has been approved for triple‐negative breast cancer (TNBC). Two distinct physiological states were found that are uncorrelated to tumor mutational burden (TMB), microsatellite instability (MSI), *PD‐L1* expression, and intrinsic cancer subtypes.

## Graphical abstract

The immune microenvironment in breast cancer is poorly understood, and consequently, the success rate of immunotherapy is low. This study demonstrates the clinical utility of a novel spatial profiling method and reveals distinct immune microenvironments in triple‐negative breast cancer (TNBC) patients that are unresolved by current clinical indicators such as tumor mutational burden (TMB), microsatellite instability (MSI), and tumor subtype.

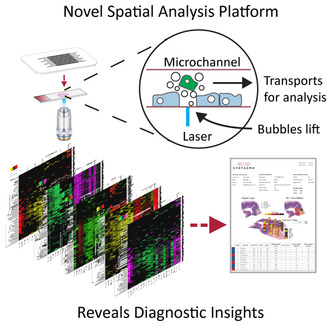

**Abbreviations**
CDx: companion diagnostic
CT: normalized cycle threshold
DAB: diaminobenzidine
FFPE: formalin‐fixed paraffin‐embedded
IHC: immunohistochemistry
ISH: *in situ* hybridization
MSI: microsatellite instability
NGS: next‐generation sequencing
PCA: principal component analysis
PCR: polymerase chain reaction
PD‐L1: programmed cell death ligand‐1
QC: quality control
ROI: region of interest
RQ: relative quantity
SCA: single‐cell analysis
TMB: tumor mutational burden
TME: tumor microenvironment
TNBC: triple‐negative breast cancer

## 1 Introduction

The role of the tumor microenvironment (TME) in immuno‐oncology has driven demand for technologies that deliver *in situ*, or spatial, molecular information [1, 2]. Traditional molecular analysis methods do not resolve compartmentalized heterogeneity, which is becoming key to predicting both acquired drug resistance to targeted therapies as well as patient response to immunotherapy [3, 4, 5, 6]. Due to its highly heterogeneous TME, triple‐negative breast cancer (TNBC) is one area of study that stands to benefit from such spatial characterization. As an aggressive subtype that represents 15–20% of all breast cancers, TNBC has a high rate of metastasis and is particularly difficult to treat due to the currently limited targeted therapeutics [7, 8]. Currently, the immunohistochemical (IHC) assessment of *PD‐L1* of immune cells (SP142 clone, Roche Tissue Diagnostics, Oro valley, AZ, USA) is the only FDA microenvironment‐based companion diagnostic test that has been approved for TNBC, serving to select *PD‐L1* positive patients for treatment with atezolizumab. This selection only increases the response rate to 23%, from around 5% without selection [9, 10].

Emerging spatial technologies improve on the staining, IHC, and *in situ* hybridization (ISH) techniques that have been in use for decades by integrating pathological imaging with multiplexed molecular analysis. However, the practicality of existing solutions limits their utility and commercial adoption. Existing platforms rely on custom chemistry, have limited multiplexing, experience low repeatability, rely on indirect measurements that introduce variability, and have difficulty in detecting targets in low abundance [11, 12].

Here, we present a novel tissue preparation method that enables any pre‐existing assay to produce spatial maps from any tissue type and with any type of molecular analysis. This method, the Molecular Fingerprint™ (mPrint™, bioSyntagma, Tempe, AZ, USA), uses microfluidics and optical cavitation to remove cells from tissue sections and indexes and transports them to a microplate where they are processed using any desired modality, such as next‐generation sequencing (NGS), polymerase chain reaction (PCR), and methylation analysis.

To demonstrate the utility of the mPrint in assessing the tumor microenvironment, spatially correlated samples were collected from primary tumors of 24 breast cancer patients (four luminal, four HER2+, and 14 TNBC samples), and analyzed using a custom qPCR panel of 248 genes. Concordance to IHC data collected from the same samples was assessed for method validation. Gene co‐expression networks were generated to identify correlations between different sample subtypes and gene pathways. Results were analyzed in the context of the patient's overall tumor mutational burden (TMB), microsatellite instability (MSI), and cancer subtype.

## 2 Materials and methods

### 2.1 Immunohistochemistry

The current study used remnant breast cancer tissue samples from 22 patients provided by Caris Life Sciences in 2019 (Phoenix, AZ, USA). Caris Life Sciences de‐identified all histopathologic reports and remnant breast cancer tissue samples from the referring laboratories. Based on this, the study complied with 45 CFR 46.101(b), was deemed exempt from Institutional Review Board (IRB) approval, and waived consent requirements. The study complied with the guidelines for human studies provided by the World Medical Association Declaration of Helsinki. IHC analysis was performed on formalin‐fixed paraffin‐embedded (FFPE) tumor samples using automated staining techniques. The primary antibody against CD3 was 2GV6 (Roche Tissue Diagnostics), anti‐CD4 was SP35 (Roche Tissue Diagnostics), anti‐CD8 was SP57 (Roche Tissue Diagnostics), anti‐CD20 was L26 (Roche Tissue Diagnostics), anti‐CD45 was LCA 2B11 & PD7/26 (Cell Marque, Rocklin, CA, USA), anti‐CD68 was KP‐1 (Roche Tissue Diagnostics), anti‐PD‐1 was NAT105 (Cell Marque), anti‐PD‐L1 was SP142 (Abcam, Cambridge, UK), and anti‐NY‐ESO‐1 was E978 (Sigma‐Aldrich, St. Louis, MO, USA).

### 2.2 Immunohistochemistry (IHC) quantification

qupath [13] (version 0.2.0‐m2, open source) and imagej (version 1.52o, open source) were used in conjunction with a color deconvolution method to quantify the amount of DAB stain present in an isolated portion of a total IHC slide [13, 14, 15]. Initially, XY coordinates were imported into qupath and aligned with isolated regions of interest (ROIs) for IHC quantification. Each individual ROI was imported into imagej with a down‐sample factor of 4. The color deconvolution method was applied per ROI by selecting pixels that related to each stain type. Stain 1: diaminobenzidine (DAB), Stain 2: hematoxylin, and Stain 3: residual (background). After the stains were selected, the ROI outline was added via the ROI manager, and then, the IHC profiler Macro was used to quantify the percentage contribution of pixels in the color‐deconvolved DAB image. The IHC profiler logged a percentage of high‐positive, positive, low‐positive, and negative pixel values as a percentage of the whole image which was then used to assign the ROI (healthy, interface, cancer, necrotic) a specific score and calculate the area of the ROI that is represented by DAB pixels. The score was set by taking the sum of 3*[high‐positive percentage], 2*[positive percentage], and 1*[low‐positive] [16]. In addition, the total percentage (high‐positive, positive, and low‐positive) of DAB pixels was multiplied by the total pixel count to find the total area of DAB‐stained pixels in the area of the ROI to supply an area vs. gene expression.

### 2.3 Microsatellite instability and tumor mutational burden measurement

Microsatellite instability (MSI) and tumor mutational burden (TMB) were assessed by NGS according to previously published methods [17, 18].

### 2.4 mPrint sample collection

Slides containing FFPE tissue samples were adhered to a LightStream FloCell™ cartridge and attached to the mPrint. Three separate tissue compartments were identified by a pathologist, consisting of (a) Normal—tissue away from the tumor, (b) Interface—area between viable tumor and inflammatory components, and (c) Cancer—viable carcinoma proper (> 90% cancer cells). ROIs were then removed using microfluidic cavitation techniques under fluid flow.

### 2.5 RNA extraction, reverse transcription, and target preamplification

RNA was extracted from collected samples using the RNeasy FFPE Kit (Qiagen, Hilden, Germany, Catalog #73504). The FFPE RNA (1 ng) was reverse transcribed using random hexamer priming in a 20 μL reaction using PrimeScript™ 1st strand cDNA Synthesis Kit (Takara Bio USA, Inc., San Jose, CA, USA, Catalog #6110A) following the manufacturer's instructions. Half (10 μL) of the cDNA reaction mix was then combined with 25 μL Prelude™ PreAmp Master Mix (Takara Bio USA, Inc., Catalog #638541) and a pool of 248 oligo pairs (400 nm each oligo; 496 total oligos) such that the target concentration of each oligo in the final 50 μL PreLude reaction was approximately 40 nm. After an initial enzyme activation step (95 °C, 2 min), the reaction was cycled for a limited number of cycles to increase the target yield 16 times (95 °C, 30 s; 50 °C, 5 min; 60 °C, 2 min), followed by a hold at 4 °C.

### 2.6 qPCR on the SmartChip™ Real‐Time PCR system

The analytical qPCR components were assembled and dispensed as described in the SmartChip® MyDesign Kit (Takara Bio USA, Inc., Catalog #640032) user manual. Briefly, the cDNA preamplification reaction mixture described above was diluted 1 : 10 with nuclease‐free water, combined with SmartChip™ TB Green® Gene Expression Master Mix (Takara Bio USA, Inc., Catalog #640211) and aliquoted into the MSND 384‐well sample source plate (Takara Bio USA, Inc., Catalog #640018). Separately, each of the 248 assay pairs was combined with SmartChip TB Green® Master Mix and aliquoted in separate wells of the assay source plate so that each of the final single‐plex reactions in the chip would be present at 300 nm in each 100 nL reaction. Samples and assays were dispensed into the 5184 well SmartChip MyDesign Chip using the SmartChip MultiSample NanoDispenser (Takara Bio USA, Inc., Catalog #640001) in a 248‐assay × 20 sample MyDesign dispense pattern. The chips were sealed with optical cycling film, spun in an Eppendorf refrigerated centrifuge (696 ***g***, 4 °C, 5 min), and then loaded into the SmartChip Real‐Time PCR Cycler (Takara Bio USA, Inc., Catalog #640023). After an initial enzyme activation step (95 °C, 3 min), the chip was cycled 35 times (95 °C, 20 s; 56 °C, 20 s; 60 °C, 15 s), followed by a melt curve program. Data were analyzed according to the standard settings in the smartchip qpcr software (Takara Bio USA, Inc.).

### 2.7 Hierarchical clustering of a single ROI

Normalized cycle threshold (*C*_T_) values were converted to relative quantity (RQ) values assuming an amplification efficiency value of 2 during on chip qPCR. Hierarchical clustering was performed on the 248 genes across all patients using two‐way Euclidean distance metrics. Clusters were evaluated by average linkage for inter‐cluster separation.

### 2.8 K‐Means clustering for ‘spatial clustering’

Building on the groups discovered by the hierarchical clustering, data from all regions were dimensionally reduced via principal component analysis (PCA) from a length of 248 genes × 3 regions = 738 gene–region pairs (e.g., *WEE1*—interface per patient to a latent vector of length 11 per region, 11 × 3 = 33). These latent vectors were clustered based on the K‐Means algorithm using Euclidean distance to ensure that discovered clusters contained all region data and compared with the previous hierarchical clustering. The K‐Means algorithm was run with varying cluster centers, from 2 to 8. Cluster stability over multiple runs and several performance metrics (Davies–Bouldin, Silhouette, and Calinski–Harabaz scoring) were used to evaluate goodness of fit. K‐Means clusters at K = 2 were discovered to be similar to single ROI hierarchical clustering (of the interface ROI) at a single‐split level.

### 2.9 Gene co‐expression visualizations

Based on RQ values across the *n* = 22 breast cancer patients, modified versions of gene co‐expression plots were created. A subset of pathways was selected for visualization: checkpoint, tumor suppressors, growth regulation, DNA repair, receptors, and T‐cell regulation. This subset of pathways was selected based on a need to simplify the amount of data being visualized, as a larger number of pathways would make reading the gene co‐expression plots impractical. Some gene–region pairs were removed from the visualization based on a missing value threshold across the patients, in order to remove the influence of correlations based on gene–region pairs that are largely null, having been removed by the qPCR quality control (QC) process but represented as an arbitrary RQ value. Correlations between gene–region pairs were then calculated based on Pearson correlation at a confidence interval of 95% or greater. Based on thresholds for positive and inverse correlations, subsets of purely intra‐correlated groups of gene–region pairs of arbitrary length in threshold margins were created. Each node color is assigned to a specific pathway, and each edge color indicates a purely intra‐correlated gene–region pair group. Shared nodes between intra‐correlated gene–region groups indicate a shared gene–region pair between groups. These central nodes shared between many groups have a higher importance as they indicate a high degree of potential causal correlations to gene–region expression levels. This process was completed for both the un‐clustered patient data and clustered/split patient data, and for differing missing value thresholds, positive correlation thresholds, and inverse correlation thresholds. These co‐expression graphs were undirected and drawn in a circular layout. Visualization of analysis was performed using python‐based Matplotlib (open source).

## 3 Results and Discussion

### 3.1 mPrint validation

**Fig. 1:**
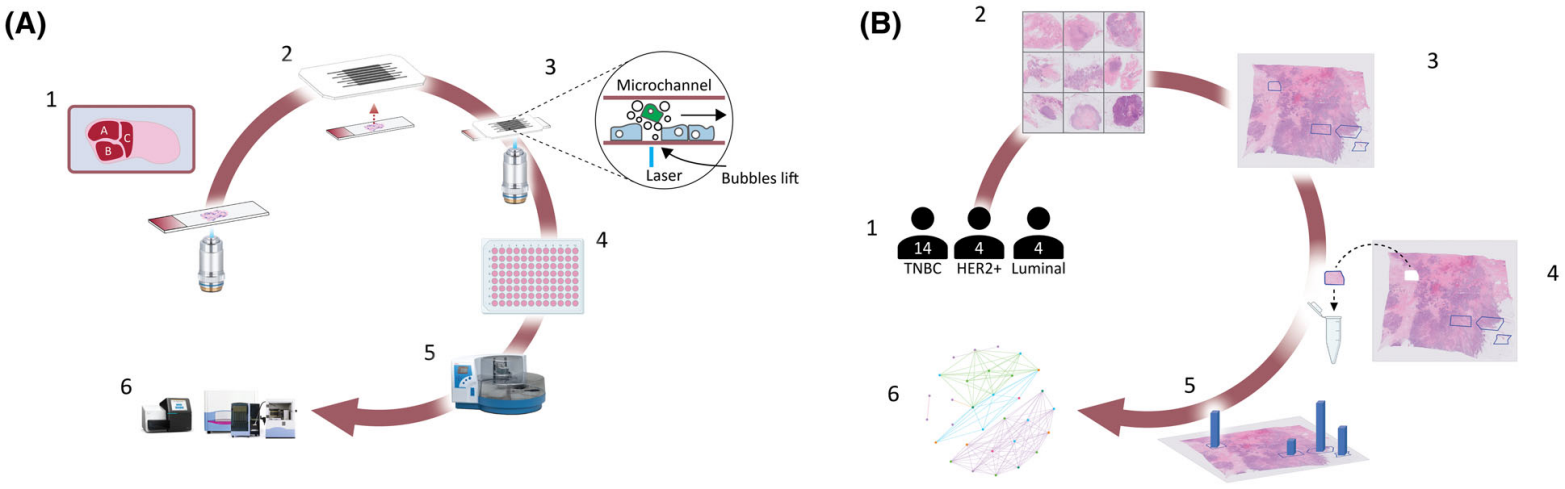
mPrint workflow and study overview. Molecular Fingerprint (mPrint) uses microfluidics and optical cavitation to remove cells from tissue sections and indexes and transports them to a microplate where they are processed using any desired modality, such as next‐generation sequencing (NGS), quantitative polymerase chain reaction (qPCR), and methylation analysis. (A1) A tissue slide is imaged and single cells or regions of interest (ROIs) are selected for removal. (A2) The tissue slide or serial section is attached to microfluidic cartridge. (A3) Laser‐induced cavitation bubbles lift cells from the slide and fluid flow transports material away. (A4) Collected material is dispensed onto a 96‐well plate, or other compatible format. (A5) DNA, RNA, or protein is isolated and (A6) material is analyzed using any commercially available assay such as NGS or qPCR. (B1) A cohort of 22 patients consisting of 14 triple‐negative breast cancer (TNBC), 4 HER2+, and 4 luminal‐like patients was identified. (B2) Tissues from each patient were analyzed using hematoxylin and eosin (H&E) and standard immunohistochemistry staining (IHC). (B3) ROIs were identified by a pathologist and imported into the system's software. (B4) ROIs were removed using optical cavitation. (B5) Material was analyzed using qPCR on a panel of 248 genes, and results were digitally reconstructed, as a map overlaid on the original tissue imagery for analysis. (B6) Correlations between gene expression and spatial location were assessed.

**Fig. 2:**
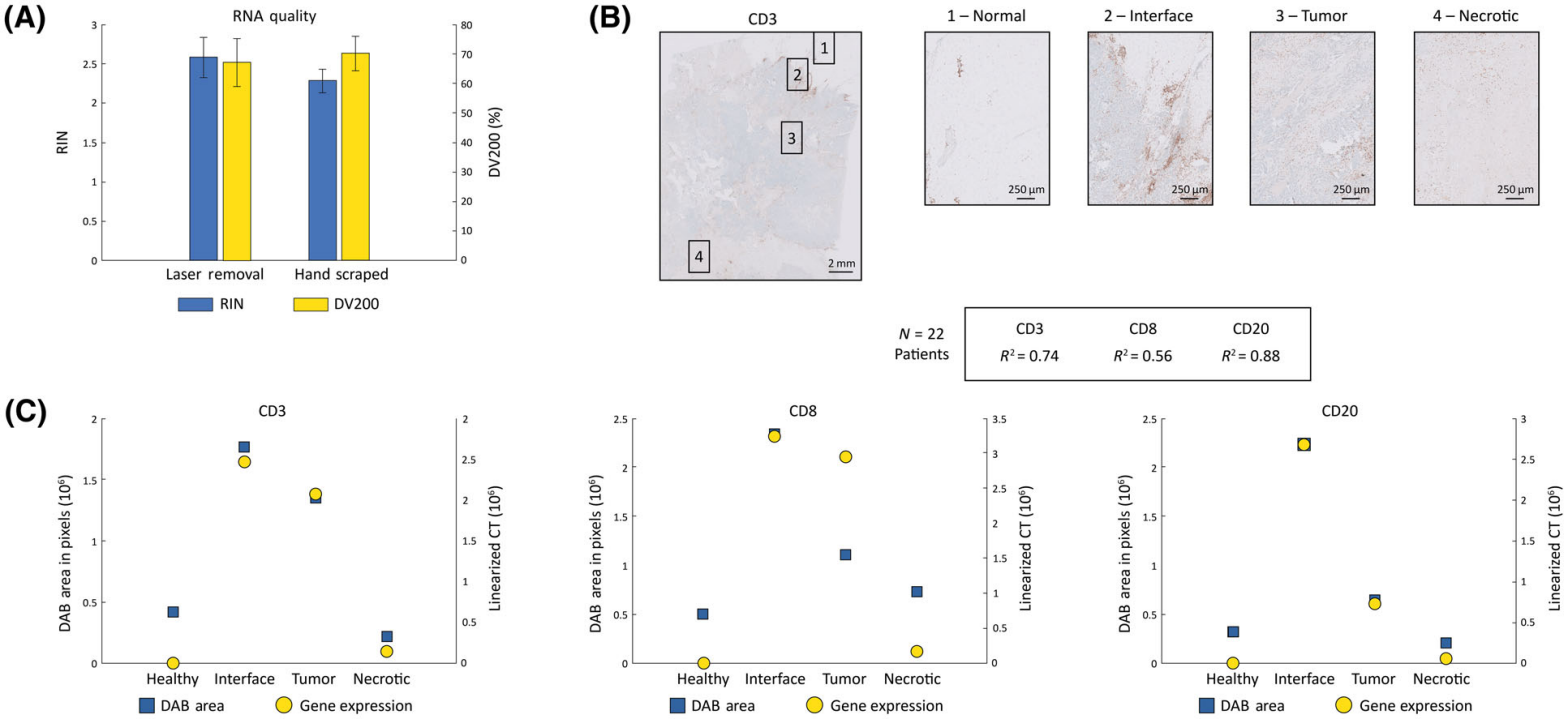
RNA quality and correlation to protein expression detected by immunohistochemistry (IHC). (A) High‐quality nucleic acids are important for successful downstream analysis by methods such as next‐generation sequencing (NGS) or quantitative polymerase chain reaction (qPCR). To evaluate the effect of optical cavitation on RNA quality, cells isolated by mPrint were compared with hand‐scraped cells directly from the tissue block, and their quality was compared. Using a bioanalyzer, the RNA integrity number (RIN) was determined to measure the overall fragmentation of nucleic acids, and the percentage of RNA fragments greater than 200 base pairs (DV200) was measured to establish its suitability for sequencing. Ten samples were tested with *N* = 5/group, and SD plotted on the graph, showing no significant differences in quality between mPrint and hand‐scraping specimen. (B) Representative whole slide and ROI images used for immunohistochemistry (IHC) quantification. Shown is CD3 expression by IHC with pathologist annotations indicating regions of healthy, interface, cancer, and necrotic tissue. The scale bar is 2 mm in the main image, and 250 μm in the image inserts. (C) Protein expression measured by IHC was correlated with gene expression of the protein‐encoding gene measured by qPCR. Overall coefficients of correlation (*R* ^2^) were determined for CD3, CD8, and CD20 across *N* = 22 patients. Representative correlative graphs are shown for each gene where protein expression is plotted by quantifying the level of diaminobenzidine (DAB) in the IHC image, and gene expression is co‐plotted by measuring linearized cycle threshold (*C*_T_) values from qPCR.

The initial goal of this study was to validate the mPrint workflow and demonstrate that samples collected using the mPrint (Fig. 1) were both of sufficient quality for downstream analysis, as well as reasonably concordant with molecular profiles measured by the current standard. To address concerns that nucleic acids might be damaged by optical cavitation and fluid exposure, whole tissue slices collected using the mPrint were compared with slices of the same tissue collected using the current industry standard of hand scraping. As measured by RNA Integrity (RIN) and DV200, there were no significant differences in quality between mPrint and hand scraping (Fig. 2A). The samples had DV200 values well above the industry standard of 30% required for reliable downstream analysis [19]. While the average RIN value of ~ 2.5 is relatively low, this is well within the range expected from FFPE tissues. Notably, the measurements are very similar regardless of the collection method. Combined, these factors indicate that the mPrint does not affect sample quality.

The next step for method validation was to demonstrate concordance of IHC quantitation with qPCR gene expression measurements collected using mPrint. Based on available IHC data, 22 samples were analyzed for CD3, CD8, and CD20 expression, and the qPCR‐IHC concordance indicated by *R* ^2^ values was 0.74 for CD3, 0.56 for CD8, and 0.88 for CD20 (Fig. 2B). These *R* ^2^ values were comparable to or higher than currently available concordance measured on an existing spatial analysis platform [20]. The same analysis was also performed on ROIs collected using the mPrint compared with the same regions isolated on IHC images (Fig. 2C). Interestingly, the samples varied greatly in expression based on location in the TME, but followed the same general pattern with each method of measurement. Notably, differences observed in each comparison may be due to differences in *z*‐axis location inherent in using serial slices, or the fact that RNA expression does not necessarily directly correlate to protein levels, in addition to possible variations introduced by the different methods. However, overall, tissue samples collected using the mPrint seem to be comparable to samples analyzed using the current industry standard.

### 3.2 Whole tissue analysis masks heterogeneity

**Fig. 3:**
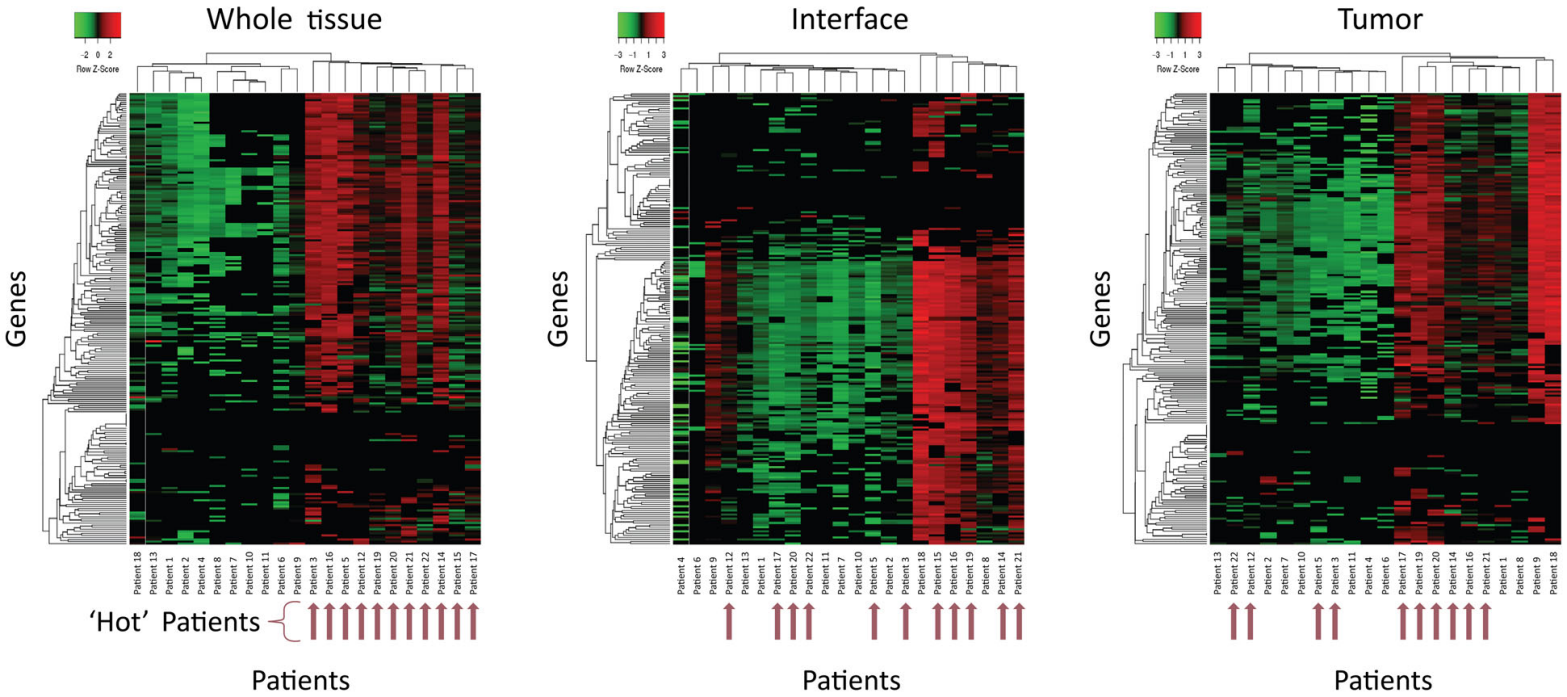
Patients cluster differently based on the tumor microenvironment (TME). A 248‐gene panel measuring gene expression in the tumor microenvironment (TME) was performed on *N* = 22 patients. Whole tissue sections of formalin‐fixed paraffin‐embedded (FFPE) tissues were analyzed in addition to pathologist‐identified regions of interest (ROI) from the tumor center and tumor interface from each patient. Hierarchical clustering is shown based on whole tissue sections (Left) compared with clustering based on only interface ROIs (Middle) and tumor ROIs (Right). Clear groups of samples with overall upregulated or downregulated gene expression are observed in the whole tissue slices, and patients considered ‘hot’ based on whole tissue analysis are designated by arrows. However, both the expression levels and patient clustering differ drastically in ROI‐specific analysis, illustrated in the redistribution of ‘hot’ patients across new clusters due to heterogeneity in the TME.

Gene expression profiles from isolated tissue compartments were compared with profiles from whole tissue scrapes from serial sections and, as expected, differences in expression profiles were identified (Fig. 3). Hierarchical clustering of the whole tissue based on the expression of the 248 gene panel stratified patients differently as compared to interface or tumor ROIs. Rather than characterizing the whole patient tumor as ‘hot’ (upregulated—up arrows in Fig. 3) or ‘cold’ (downregulated), each compartment within the resected tissue was uniquely characterized as hot/cold within the same patient. Since the current method of evaluating patients for atezolizumab treatment of TNBC based on overall *PD‐1/PD‐L1* expression is considerably ineffective, we propose that spatial molecular profiles should be evaluated for the improvement of stratifying patients more accurately according to their likelihood of treatment response.

### 3.3 Gene expression at the tumor interface does not group by tumor mutational burden, microsatellite instability, or subtype

**Fig. 4:**
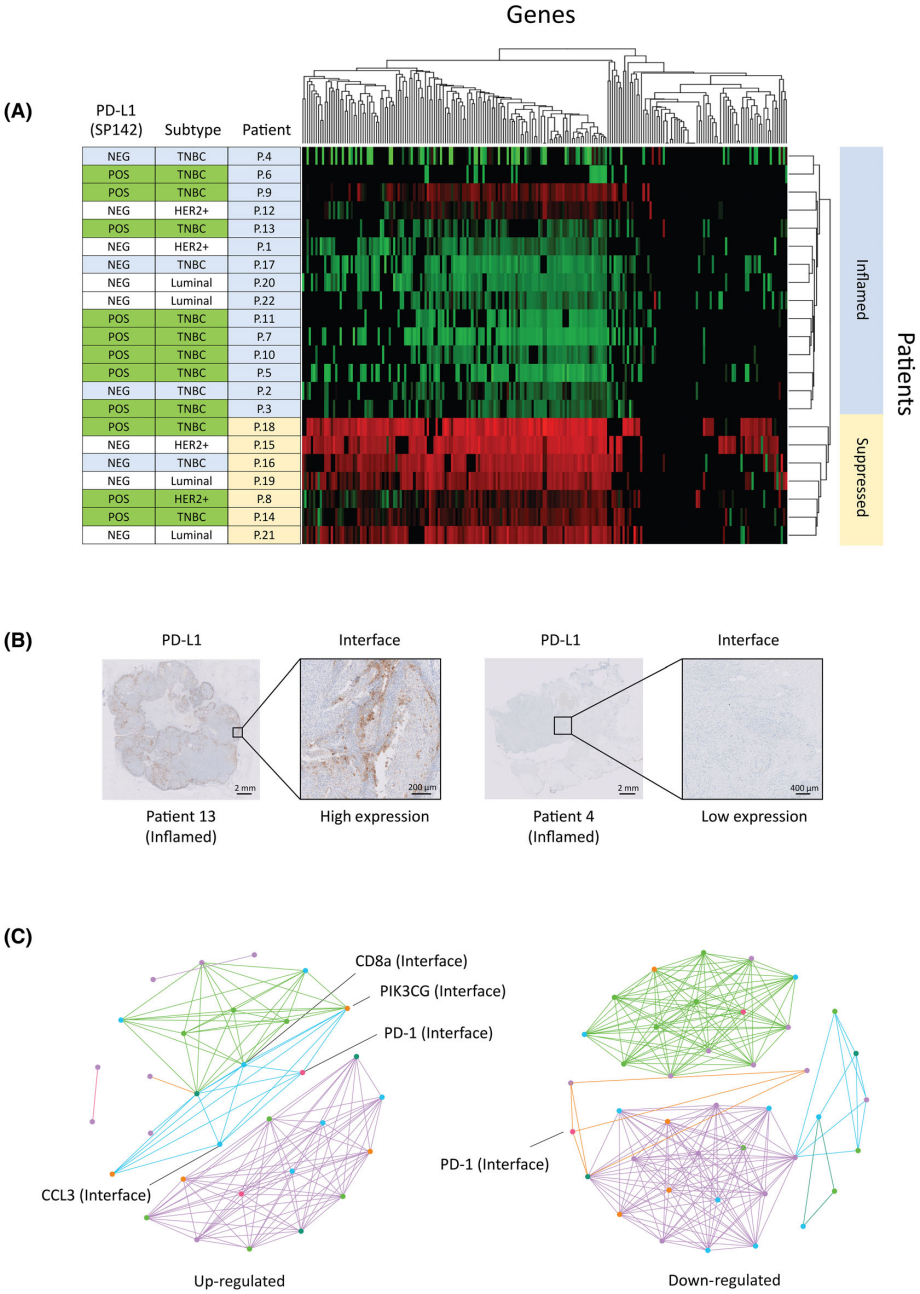
Spatial clustering analysis identifies correlations unrelated to tumor subtype or PD‐1/PD‐L1 expression. (A) Hierarchical clustering analysis from a 248‐gene panel performed on interface regions of the tumor microenvironment (TME) on *N* = 22 patients reveals two main subgroups with overall gene expression upregulation or downregulation. Samples were also assessed for molecular subtype and programmed death ligand 1 (PD‐L1) expression as determined by immunohistochemistry (IHC). (B) Patients within the same upregulated subgroup demonstrated both high and low expression of PD‐L1 as measured by IHC. The scale bar for patient 13 is 2 mm, and the image insert is 200 μm. The scale bar for patient 4 is 2 mm, and its insert is 400 um. (C) Gene co‐expression networks for up‐ and downregulated subgroups visualizing correlations between genes and their TME compartments (healthy, interface, tumor, necrotic). In the diagram, each node represents a gene from a TME compartment (gene–region pair), the node color indicates the gene pathway it belongs to, lines indicate correlations between gene–region pairs (positive correlation greater than *R* ^2^ = 0.7 or inverse correlations greater than *R* ^2^ = 0.5), and line colors indicate purely intra‐correlated groups. Shared nodes between groups indicate a shared gene–region pair between groups. In the upregulated cohort, programmed cell death 1 (PD‐1) in the tumor interface correlates to expression of other immune genes, while in the downregulated cohort no such correlations are present.

In addition to *PD‐1/PD‐L1* analysis, cancer subtype, tumor mutational burden, and microsatellite instability are all factors that are currently taken into account when making treatment recommendations. However, patient grouping by hierarchical clustering (Fig. 4A) did not clearly correlate with any of these factors, indicating that spatial analysis may be another variable to take under consideration for improved patient stratification. The tumor interface was of particular interest because of its insight into the inflammatory and immune response, and the hierarchical clustering of patients based on gene expression in the interface revealed two strongly distinct cohorts of hot and cold microenvironments that were not correlated to the patient's overall TMB, MSI, or cancer subtype, as shown in the ‘Inflamed’ and ‘Suppressed’ categories shown in Fig. 4A,B.

Modified gene co‐expression networks (Fig. 4C) were generated for each patient group to visualize the correlations between genes and their compartments for the pathways of DNA repair, checkpoint, growth regulation, tumor suppressors, T‐cell regulation, and receptors. Many genes were correlated not only to other genes in their own compartment but also to genes in adjacent compartments. These relationships may prove useful in discovering biomarkers consisting of network signatures rather than single gene relationships. For example, in the cohort of patients with upregulated microenvironments, *PD‐1* in the tumor interface was correlated (*R* = 0.83) to the expression of CD8a, as well as other genes involved in immune response *PIK3CG* (*R* = 0.74) and CCL3 (*R* = 0.96). However, in the downregulated patient group, *PD‐1* expression in the interface did not correspond to any other genes involved in the immune response. This could represent an area for future study and possible targets for immunotherapy screening beyond the currently used *PD‐L1*.

### 3.4 Limitations and future directions

While the mPrint method presents a novel approach to spatial profiling with promising applications, it is important to acknowledge its limitations. Spatial technologies are generally characterized by their degree of multiplexing (measured by the number of targets or ‘omics measured and their resolution (sub‐cellular vs. cellular). The mPrint is a high‐plex, cellular‐resolution technology. However, the current embodiment of its microfluidic design is not ideally suited for high‐throughput single‐cell analysis (SCA), where thousands or millions of cells are individually analyzed. As resolution requirements for spatial technologies vary by application, the mPrint is currently more compatible with translational and clinical applications, rather than discovery efforts that commonly employ high‐throughput SCA.

Looking ahead, the design of this novel platform lends itself to integration with other methods like auto‐stainers and molecular tools such as sequencers to provide automated sample‐to‐results solutions. Beyond tissue collection, the unique results generated by the mPrint function as an ideal input for novel informatics approaches as well. It is currently unknown what spatial resolution is necessary to extract clinically relevant information from patient samples. Ongoing preliminary studies by the authors using machine learning and generative AI have demonstrated promise in extracting clinically relevant markers from high‐resolution spatial data sets and identifying those same clinical markers in low‐resolution data sets such as those produced by the mPrint. High‐resolution datasets are costly to generate, typically only performed on physically small specimens, and computationally expensive to analyze since their files range from gigabytes to terabytes in size for a single specimen. Further development of these AI/ML methods would make companion diagnostic (CDx) development less costly and more readily translated to the clinic.

## 4 Conclusions

The mPrint represents an effective, innovative approach to gaining spatially dependent insights such as the compartmentalized heterogeneity within the tumor microenvironment. Building on this study to narrow down prognostic indicators will be a focus of future work, as the patients in this study were still undergoing treatment at the time of this work, and consequently, outcome data is not currently available to determine which microenvironment factors contribute to therapeutic response. In the meantime, results from this study suggest that patient stratification based on molecular characteristics of the tumor microenvironment differs from existing methods of patient stratification that rely on molecular subtyping of the entire tumor and has the potential as an additional variable to improve patient treatment selection.

## Conflict of interest

The authors declare no conflict of interest.

## Author contributions

CZ, AM, AF, ZG, DR, and DND conceived and designed the project. CZ, AM, SA, PM, EC, BW, ZG, DR, and DND acquired the data. CZ, AM, PM, IS, CD, AF, SV, ZG, DR, and DND analyzed and interpreted the data. CZ, SA, PM, CD, SV, DR wrote the paper. All authors contributed to the manuscript review.

## Data Availability

The data that support the findings of this study are available upon reasonable request from the corresponding author.
